# Supplementary material for: Drug repurposing for aging research using model organisms
Source: Aging Cell. 2017 Jun 16;16(5):1006–15. doi: 10.1111/acel.12626 (PMC5595691; doi:10.1111/acel.12626)
Supplement: Supplementary file 7 — Data S1 Zip‐Archive of all report cards. [file ACEL-16-1006-s007.zip › RC_GSH.pdf]

GSH

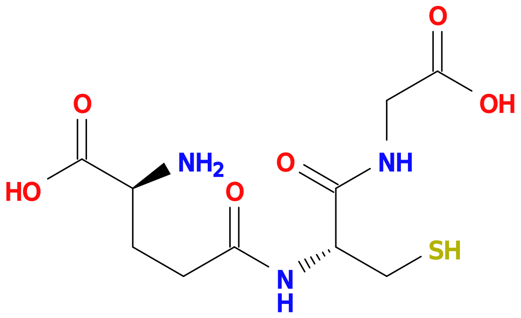

Database identifiers

ChEMBLCompound	CHEMBL1543
DrugBank	DB00143
CHEBI	16856
CHEBI	60836
eMolecules	515159

Ranking

	Rank	Score
Drosophila	321/697	0.486
C. elegans	521/591	0.055

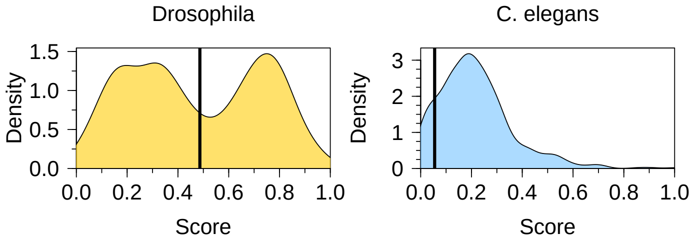

	Ageing implication	Domain conservation	Binding site conservation	Binding affinity	Bioavailability	Lipinski	Promiscuity	Purchasability	Drug approval	**Total**
Drosophila	1.0	0.982	1.0	0.634	(0.9)	-0.05	-0.199	0.1	0.075	0.486
C. elegans	1.0	0.982	1.0	0.634	0.207	-0.05	-0.199	0.1	0.075	0.055

Names

- **phytochelatin**
- Glutathione

Roles

chelator, metabolite

Status

Approved drug (according to ChEMBL)	No
Classification (according to DrugBank)	approved, nutraceutical
Number of Rule of 5 violations	1
Binding affinity to original target in log units (RF-Score prediction)	5.55
Burns *C. elegans* bioavailability prediction	-6.73

Medical Information

Indicatation: For nutritional supplementation, also for treating dietary shortage or imbalance
Mechanism of action: Glutathione (GSH) participates in leukotriene synthesis and is a cofactor for the enzyme glutathione peroxidase. It is also important as a hydrophilic molecule that is added to lipophilic toxins and waste in the liver during biotransformation before they can become part of the bile. Glutathione is also needed for the detoxification of methylglyoxal, a toxin produced as a by-product of metabolism. This detoxification reaction is carried out by the glyoxalase system. Glyoxalase I catalyzes the conversion of methylglyoxal and reduced glutathione to S-D-Lactoyl-glutathione. Glyoxalase II catalyzes the conversion of S-D-Lactoyl Glutathione to Reduced Glutathione and D-lactate. GSH is known as a cofactor in both conjugation reactions and reduction reactions, catalyzed by glutathione S-transferase enzymes in cytosol, microsomes, and mitochondria. However, it is capable of participating in non-enzymatic conjugation with some chemicals, as it is hypothesized to do to a significant extent with n-acetyl-p-benzoquinone imine (NAPQI), the reactive cytochrome P450 reactive metabolite formed by toxic overdose of acetaminophen. Glutathione in this capacity binds to NAPQI as a suicide substrate and in the process detoxifies it, taking the place of cellular protein sulfhydryl groups which would otherwise be toxically adducted. The preferred medical treatment to an overdose of this nature, whose efficacy has been consistently supported in literature, is the administration (usually in atomized form) of N-acetylcysteine, which is used by cells to replace spent GSSG and allow a usable GSH pool.
Toxicity: ORL-MUS LD50 5000 mg/kg, IPR-MUS LD50 4020 mg/kg, SCU-MUS LD50 5000 mg/kg, IVN-RBT LD50 > 2000 mg/kg, IMS-MUS LD50 4000 mg/kg
Retail: Letco Medical Inc., Medisca Inc., Spectrum Pharmaceuticals
L-glutathione powder - 3.0 USD / g
Glutathione-l reduced powder - 10.71 USD / g
Glutathione crystals - 21.32 USD / g

(Information from DrugBank)

Compound Target Characteristics

Chloride intracellular channel protein 1

Best gene implication in ageing for this target family came from gene Q9VY78 via mapping the annotation from FlyBase FBgn0030529 annotated in FlyBase 2014-03-11. Annotation GO 8340 (determination of adult lifespan) was Inferred from Mutant Phenotype

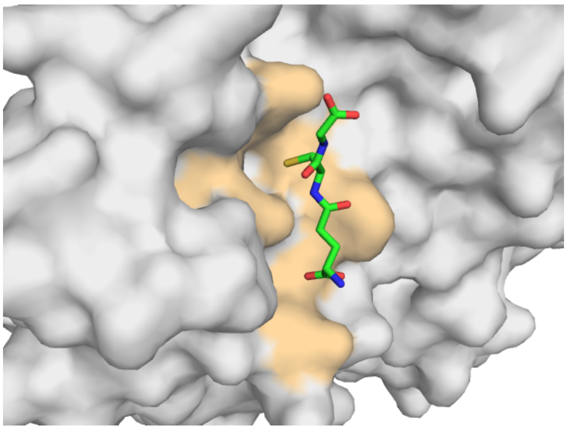

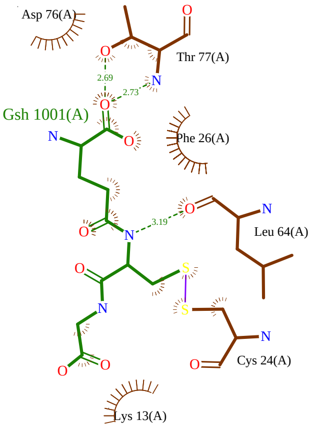

protein	amino acids contacts (binding site)
PDB:1k0n:chainA:O00299	K C F L D T
tr:Q5SRT3:Q5SRT3_HUMAN	K C F L D T
sp:O00299:CLIC1_HUMAN	K C F L D T
sp:Q6MG61:CLIC1_RAT	K C F L D T
sp:Q9Z1Q5:CLIC1_MOUSE	K C F L D T
tr:Q542F1:Q542F1_MOUSE	K C F L D T
tr:Q9VY78:Q9VY78_DROME	K C F P E N
sp:Q8WQA4:EXC4_CAEEL	K D F P D N

	whole protein		domain-based		contact-based	
protein	ident	simil	ident	simil	ident	simil
PDB:1k0n:chainA:O00299	1.0	1.0	0.99	1.0	1.0	1.0
tr:Q5SRT3:Q5SRT3_HUMAN	1.0	1.0	1.0	1.0	1.0	1.0
sp:O00299:CLIC1_HUMAN	1.0	1.0	1.0	1.0	1.0	1.0
sp:Q6MG61:CLIC1_RAT	0.99	1.0	1.0	1.0	1.0	1.0
sp:Q9Z1Q5:CLIC1_MOUSE	0.98	1.0	1.0	1.0	1.0	1.0
tr:Q542F1:Q542F1_MOUSE	0.98	1.0	1.0	1.0	1.0	1.0
tr:Q9VY78:Q9VY78_DROME	0.2	0.56	0.28	0.69	0.5	0.68
sp:Q8WQA4:EXC4_CAEEL	0.19	0.5	0.26	0.64	0.5	0.51

Clic (FBgn0030529) associated phenotypes

gravitaxis defective, heat sensitive, partially lethal - majority die

(Information from FlyBase)

exc-4 (WBGene00001365) associated phenotypes

excretory canal morphology variant, lethal, locomotion variant, pale

(Information from WormBase)

exc-4 (UniProt:Q8WQA4) annotation

Function: May insert into membranes and form chloride ion channels. Involved in the formation of the excretory canal. Required to prevent cystic lumenal expansions in the excretory cell. Not required for formation of the initial tube, but is required for regulating the size of the tube lumen as it grows. (PubMed:17985355).
Subunit: Monomer. (PubMed:17985355).
Subcellular location: Cytoplasm. Membrane ; Single- pass membrane protein Note=Exists both as soluble cytoplasmic protein and as membrane protein with probably a single transmembrane domain. Localizes to various tubular membranes in distinct cell types, including the lumenal membrane of the excretory tubes.
Tissue specificity: Expressed in the secretory system, hypodermis, vulva, pharyngeal muscle, rectal gland, tubular rectal epithelium cells, and tubular neuronal support cells in the head and tail. (PubMed:14684823).
Domain: The N-terminal part (1-55) is necessary and sufficient for translocation from the cytosol to the membrane. (PubMed:14684823).
Domain: Members of this family may change from a globular, soluble state to a state where the N-terminal domain is inserted into the membrane and functions as chloride channel. A conformation change of the N-terminal domain is thought to expose hydrophobic surfaces that trigger membrane insertion (By similarity).

(Information from UniProt)

Chloride intracellular channel protein 2

Best gene implication in ageing for this target family came from gene Q9VY78 via mapping the annotation from FlyBase FBgn0030529 annotated in FlyBase 2014-03-11. Annotation GO 8340 (determination of adult lifespan) was Inferred from Mutant Phenotype

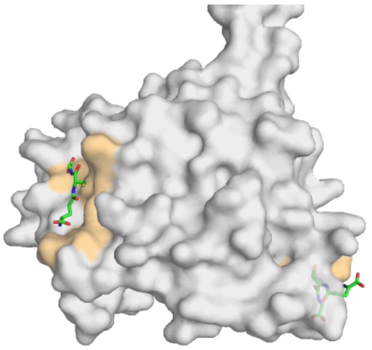

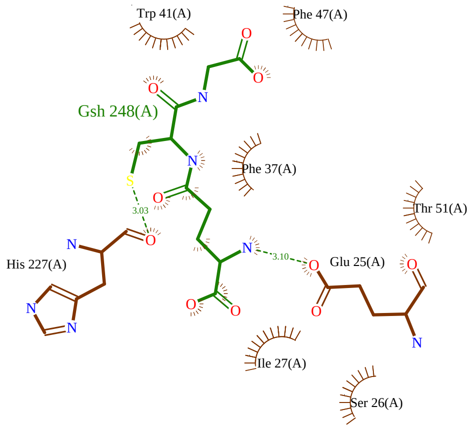

protein	amino acids contacts (binding site)										
PDB:2r4v:chainA:O15247	E	S	I	F	W	F	T	A	N	F	H
sp:O15247:CLIC2_HUMAN	E	S	I	F	W	F	T	A	N	F	H
sp:Q5M883:CLIC2_RAT	E	S	I	F	W	F	T	A	N	F	H
tr:Q543N5:Q543N5_MOUSE	E	S	I	F	W	F	T	A	A	L	N
sp:Q9QYB1:CLIC4_MOUSE	E	S	I	F	W	F	T	A	A	L	N
sp:Q8BHB9:CLIC6_MOUSE	E	S	I	F	W	F	T	A	I	Y	N
tr:Q9VY78:Q9VY78_DROME	R	R	K	F	Y	L	T	-	A	K	Q
sp:Q8WQA4:EXC4_CAEEL	R	R	I	W	Y	V	T	Q	V	H	E

	whole protein		domain-based		contact-based	
protein	ident	simil	ident	simil	ident	simil
PDB:2r4v:chainA:O15247	1.0	1.0	1.0	1.0	1.0	1.0
sp:O15247:CLIC2_HUMAN	1.0	1.0	1.0	1.0	1.0	1.0
sp:Q5M883:CLIC2_RAT	0.92	0.97	0.94	0.98	1.0	1.0
tr:Q543N5:Q543N5_MOUSE	0.6	0.83	0.67	0.91	0.73	0.81
sp:Q9QYB1:CLIC4_MOUSE	0.6	0.83	0.67	0.91	0.73	0.81
sp:Q8BHB9:CLIC6_MOUSE	0.27	0.37	0.66	0.88	0.73	0.78
tr:Q9VY78:Q9VY78_DROME	0.27	0.6	0.32	0.7	0.18	0.4
sp:Q8WQA4:EXC4_CAEEL	0.2	0.52	0.24	0.62	0.18	0.55

Clic (FBgn0030529) associated phenotypes

gravitaxis defective, heat sensitive, partially lethal - majority die

(Information from FlyBase)

exc-4 (WBGene00001365) associated phenotypes

excretory canal morphology variant, lethal, locomotion variant, pale

(Information from WormBase)

exc-4 (UniProt:Q8WQA4) annotation

Function: May insert into membranes and form chloride ion channels. Involved in the formation of the excretory canal. Required to prevent cystic lumenal expansions in the excretory cell. Not required for formation of the initial tube, but is required for regulating the size of the tube lumen as it grows. (PubMed:17985355).

Subunit: Monomer. (PubMed:17985355).

Subcellular location: Cytoplasm. Membrane ; Single- pass membrane protein Note=Exists both as soluble cytoplasmic protein and as membrane protein with probably a single transmembrane domain. Localizes to various tubular membranes in distinct cell types, including the lumenal membrane of the excretory tubes.

Tissue specificity: Expressed in the secretory system, hypodermis, vulva, pharyngeal muscle, rectal gland, tubular rectal epithelium cells, and tubular neuronal support cells in the head and tail. (PubMed:14684823).

Domain: The N-terminal part (1-55) is necessary and sufficient for translocation from the cytosol to the membrane. (PubMed:14684823).

Domain: Members of this family may change from a globular, soluble state to a state where the N-terminal domain is inserted into the membrane and functions as chloride channel. A conformation change of the N-terminal domain is thought to expose hydrophobic surfaces that trigger membrane insertion (By similarity).

(Information from UniProt)

Hematopoietic prostaglandin D synthase

Best gene implication in ageing for this target family came from gene A4UZL5 via mapping the annotation from Ensembl FBgn0010226 via mapping the annotation from EntrezGene 36927 via mapping the annotation from GenAgeModels 0851 annotated in GenAge release 17.

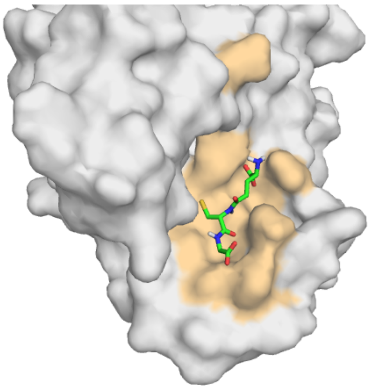

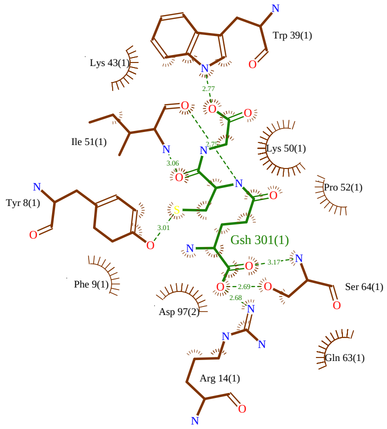

protein	amino acids contacts (binding site)										
PDB:1pd2:chain1:O35543	Y	F	R	W	K	K	I	P	Q	S	D
sp:O60760:HPGDS_HUMAN	Y	F	R	W	K	K	I	P	Q	S	D
sp:O35543:HPGDS_RAT	Y	F	R	W	K	K	I	P	Q	S	D
sp:Q9JHF7:HPGDS_MOUSE	Y	F	R	W	K	K	I	P	Q	S	D
tr:A4UZL5:A4UZL5_DROME	Y	F	L	W	K	Q	M	P	Q	S	D
sp:P41043:GST1_DROME	Y	F	L	W	K	Q	M	P	Q	S	D
tr:Q21945:Q21945_CAEEL	Y	F	R	W	K	K	L	P	Q	S	D

	whole protein		domain-based		contact-based	
protein	ident	simil	ident	simil	ident	simil
PDB:1pd2:chain1:O35543	1.0	1.0	1.0	1.0	1.0	1.0
sp:O60760:HPGDS_HUMAN	0.81	0.94	0.81	0.94	1.0	1.0
sp:O35543:HPGDS_RAT	1.0	1.0	1.0	1.0	1.0	1.0
sp:Q9JHF7:HPGDS_MOUSE	0.94	0.98	0.94	0.98	1.0	1.0
tr:A4UZL5:A4UZL5_DROME	0.32	0.64	0.32	0.64	0.73	0.85
sp:P41043:GST1_DROME	0.32	0.64	0.32	0.64	0.73	0.85
tr:Q21945:Q21945_CAEEL	0.38	0.76	0.38	0.76	0.91	1.0

GstS1 (FBgn0010226) associated phenotypes

partially lethal - majority live

(Information from FlyBase)

GstS1 (UniProt:P41043) annotation

Function: Conjugation of reduced glutathione to a wide number of exogenous and endogenous hydrophobic electrophiles. May be involved in the detoxification of metabolites produced during cellular division and morphogenesis.
Subunit: Homodimer. ECO:0000269—PubMed:12547198.
Developmental stage: Expressed throughout development with highest levels being observed in nonfeeding stages, i.e. During embryonic and pupal development.

(Information from UniProt)

Glutathione reductase, mitochondrial

Best gene implication in ageing for this target family came from gene P91938 annotated in UniProt release 2014_02. Annotation GO 8340 (determination of adult lifespan) was Inferred from Mutant Phenotype

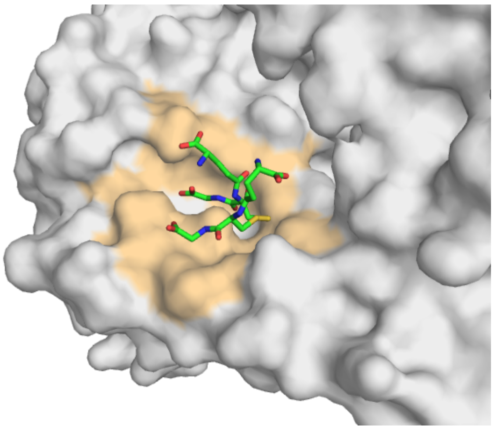

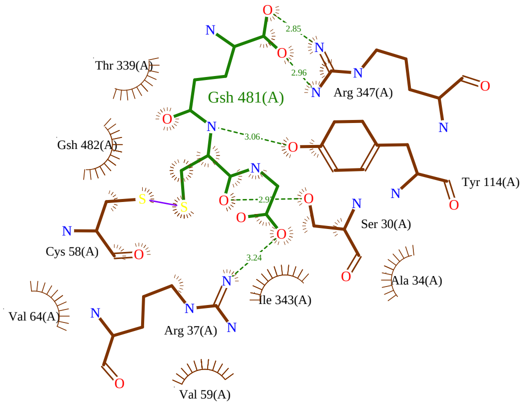

protein	amino acids contacts (binding site)												
PDB:1dnc:chainA:P00390	S	A	R	C	V	V	L	I	Y	N	T	I	R
PDB:1gre:chainA:P00390	S	A	R	C	V	V	L	I	Y	N	T	I	R
PDB:1gsn:chainA:P00390	S	A	R	C	V	V	L	I	Y	N	T	I	R
sp:P00390:GSHR_HUMAN	S	A	R	C	V	V	L	I	Y	N	T	I	R
tr:F1LRE1:F1LRE1_RAT	-	-	-	-	-	-	L	I	Y	N	T	I	R
sp:P47791:GSHR_MOUSE	S	A	R	C	V	V	L	I	Y	N	T	I	R
sp:P91938:TRXR1_DROME	S	A	K	C	V	I	V	V	T	D	T	V	R
tr:Q86DB5:Q86DB5_CAEEL	S	A	R	C	V	V	L	L	Y	G	T	I	R
tr:Q93379:Q93379_CAEEL	S	A	R	C	V	V	L	L	Y	G	T	I	R
sp:P41921:GSHR_YEAST	S	A	R	C	V	V	L	I	Y	N	T	I	R

	whole protein		domain-based		contact-based	
protein	ident	simil	ident	simil	ident	simil
PDB:1dnc:chainA:P00390	1.0	1.0	1.0	1.0	1.0	1.0
PDB:1gre:chainA:P00390	1.0	1.0	1.0	1.0	1.0	1.0
PDB:1gsn:chainA:P00390	0.99	0.99	1.0	1.0	1.0	1.0
sp:P00390:GSHR_HUMAN	1.0	1.0	1.0	1.0	1.0	1.0
tr:F1LRE1:F1LRE1_RAT	0.68	0.76	0.62	0.72	0.54	0.0
sp:P47791:GSHR_MOUSE	0.8	0.89	0.86	0.96	1.0	1.0
sp:P91938:TRXR1_DROME	0.28	0.64	0.36	0.73	0.46	0.82
tr:Q86DB5:Q86DB5_CAEEL	0.51	0.74	0.6	0.86	0.85	0.93
tr:Q93379:Q93379_CAEEL	0.5	0.73	0.6	0.86	0.85	0.93
sp:P41921:GSHR_YEAST	0.44	0.73	0.49	0.77	1.0	1.0

Trxr-1 (FBgn0020653) associated phenotypes

aging defective, developmental rate defective, lethal - all die before end of P-stage, lethal - all die before end of pupal stage, neuroanatomy defective, partially, partially lethal - majority die, some die during pharate adult stage, some die during pupal stage, stress response defective

(Information from FlyBase)

Trxr-1 (UniProt:P91938) annotation

Function: Thioredoxin system is a major player in glutathione metabolism, due to the demonstrated absence of a glutathione reductase. Functionally interacts with the Sod/Cat reactive oxidation species (ROS) defense system and thereby has a role in preadult development and life span. Lack of a glutathione reductase suggests antioxidant defense in Drosophila, and probably in related

insects, differs fundamentally from that in other organisms. (PubMed:11158675, PubMed:11525742, PubMed:11796729).

Cofactor: FADNote=Binds 1 FAD per subunit.;

Biophysicochemical properties: Kinetic parameters: KM=6.5 uM for NADPH (at pH 7.4) (PubMed:11158675, PubMed:11782468, PubMed:11796729, PubMed:11877442, PubMed:17385893, PubMed:18211101, PubMed:18991392); KM=1 uM for NADPH (at pH 7.4, 2 mM EDTA, 100 mM KPO(4)) (PubMed:11158675, PubMed:11782468, PubMed:11796729, PubMed:11877442, PubMed:17385893, PubMed:18211101, PubMed:18991392); KM=1 uM for NADPH (isoform B at pH 7.4, 2 mM EDTA, 100 mM KPO(4)) (PubMed:11158675, PubMed:11782468, PubMed:11796729, PubMed:11877442, PubMed:17385893, PubMed:18211101, PubMed:18991392); KM=7.0 uM for dhd (at pH 7.4, 200 uM NADPH, 100 mM KPO(4)) (PubMed:11158675, PubMed:11782468, PubMed:11796729, PubMed:11877442, PubMed:17385893, PubMed:18211101, PubMed:18991392); KM=141 uM for dhd (at pH 7.0, 0.15 mM NADPH, 1 mM EDTA, 1 mg/ml insulin, 50 mM KPO(4), 25 degrees Celsius) (PubMed:11158675, PubMed:11782468, PubMed:11796729, PubMed:11877442, PubMed:17385893, PubMed:18211101, PubMed:18991392); KM=7 uM for dhd (at pH 7.4, 2 mM EDTA, 100 mM KPO(4)) (PubMed:11158675, PubMed:11782468, PubMed:11796729, PubMed:11877442, PubMed:17385893, PubMed:18211101, PubMed:18991392); KM=19 uM for dhd (isoform B at pH 7.4, 2 mM EDTA, 100 mM KPO(4)) (PubMed:11158675, PubMed:11782468, PubMed:11796729, PubMed:11877442, PubMed:17385893, PubMed:18211101, PubMed:18991392); KM=5.9 uM for Trx-2 (at pH 7.4, 100 uM NADPH, 2 mM EDTA, 100 mM KPO(4)) (PubMed:11158675, PubMed:11782468, PubMed:11796729, PubMed:11877442, PubMed:17385893, PubMed:18211101, PubMed:18991392); KM=310 uM for 5,5'-dithiobis-2-nitrobenzoic acid (DTNB) (at pH 7.4, 100 uM NADPH, 100 mM KPO(4)) (PubMed:11158675, PubMed:11782468, PubMed:11796729, PubMed:11877442, PubMed:17385893, PubMed:18211101, PubMed:18991392); KM=0.17 mM for DTNB (at pH 7.0, 0.2 mM NADPH, 10 mM EDTA, 100 mM KPO(4), 25 degrees Celsius) (PubMed:11158675, PubMed:11782468, PubMed:11796729, PubMed:11877442, PubMed:17385893, PubMed:18211101, PubMed:18991392); KM=380 uM for DTNB (at pH 7.4, 2 mM EDTA, 100 mM KPO(4)) (PubMed:11158675, PubMed:11782468, PubMed:11796729, PubMed:11877442, PubMed:17385893, PubMed:18211101, PubMed:18991392); KM=410 uM for DTNB (isoform B at pH 7.4, 2 mM EDTA, 100 mM KPO(4)) (PubMed:11158675, PubMed:11782468, PubMed:11796729, PubMed:11877442, PubMed:17385893, PubMed:18211101, PubMed:18991392); KM=675 uM for methylseleninate (100 uM NADPH) (PubMed:11158675, PubMed:11782468, PubMed:11796729, PubMed:11877442, PubMed:17385893, PubMed:18211101, PubMed:18991392); Vmax=24.3 umol/min/mg enzyme toward NADPH (at pH 7.4) (PubMed:11158675, PubMed:11782468, PubMed:11796729, PubMed:11877442, PubMed:17385893, PubMed:18211101, PubMed:18991392); Vmax=16 umol/min/mg enzyme toward Trx-2 (at pH 7.4, 100 uM NADPH, 2 mM EDTA, 100 mM KPO(4), 25 degrees Celsius) (PubMed:11158675, PubMed:11782468, PubMed:11796729, PubMed:11877442, PubMed:17385893, PubMed:18211101, PubMed:18991392); Note=Measurements were conducted with isoform A unless noted otherwise.; pH dependence: Optimum pH is 7.6 for isoform A with Trx-2 and NADPH as substrates. (PubMed:11158675, PubMed:11782468, PubMed:11796729, PubMed:11877442, PubMed:17385893, PubMed:18211101, PubMed:18991392);

Subunit: Homodimer. (PubMed:17385893).

Subcellular location: Isoform B: Mitochondrion.

Subcellular location: Isoform A: Cytoplasm.

Tissue specificity: During embryogenesis, expression is seen in germ cell progenitors, developing midgut, hindgut and proventriculus. (PubMed:11525742).

Developmental stage: Expressed both maternally and zygotically during all stages of development, highest expression during adult stages. (PubMed:11525742, PubMed:11796729).

Miscellaneous: The active site is a redox-active disulfide bond.

Caution: Was originally thought to be a glutathione reductase. (PubMed:9056265).

(Information from UniProt)

gsr-1 (WBGene00008117) associated phenotypes
lethal, sterile

(Information from WormBase)

Glutathione S-transferase P 1

Best gene implication in ageing for this target family came from gene A8MX94 via mapping the annotation from Ensembl ENSG00000084207 via mapping the annotation from EntrezGene 2950 via mapping the annotation from GenAgeHuman 0157 annotated in GenAge release 17.

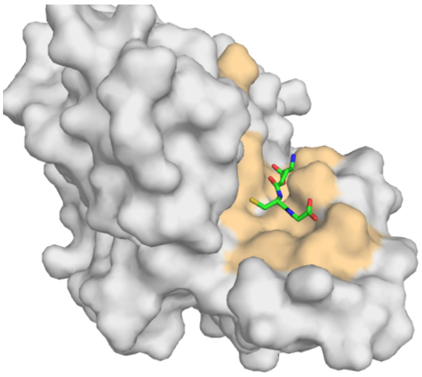

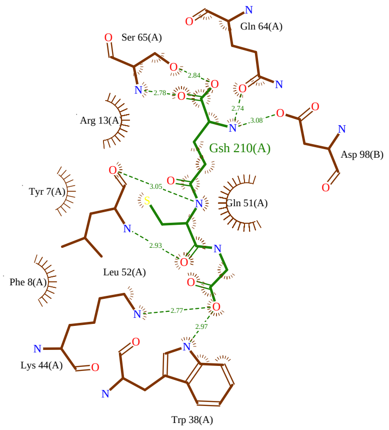

protein	amino acids contacts (binding site)
PDB:1gsy:chainA:P19157	Y F R W K Q L Q S D
tr:A8MX94:A8MX94_HUMAN	Y F R W K Q L Q S D
sp:P09211:GSTP1_HUMAN	Y F R W K Q L Q S D
tr:B6DYQ7:B6DYQ7_RAT	Y F R W K Q L Q S D
sp:P04906:GSTP1_RAT	Y F R W K Q L Q S D
sp:P46425:GSTP2_MOUSE	Y F R W K Q L Q S D
sp:P19157:GSTP1_MOUSE	Y F R W K Q L Q S D
tr:P91505:P91505_CAEEL	Y F L W K Q L Q S D

	whole protein		domain-based		contact-based	
protein	ident	simil	ident	simil	ident	simil
PDB:1gsy:chainA:P19157	1.0	1.0	1.0	1.0	1.0	1.0
tr:A8MX94:A8MX94_HUMAN	0.7	0.79	0.69	0.78	1.0	1.0
sp:P09211:GSTP1_HUMAN	0.85	0.96	0.85	0.96	1.0	1.0
tr:B6DYQ7:B6DYQ7_RAT	0.92	0.98	0.92	0.98	1.0	1.0
sp:P04906:GSTP1_RAT	0.92	0.98	0.92	0.98	1.0	1.0
sp:P46425:GSTP2_MOUSE	0.97	0.99	0.97	0.99	1.0	1.0
sp:P19157:GSTP1_MOUSE	1.0	1.0	1.0	1.0	1.0	1.0
tr:P91505:P91505_CAEEL	0.39	0.79	0.38	0.79	0.9	0.91

Filamin-A

Best gene implication in ageing for this target family came from gene Q9VEN1 annotated in UniProt release 2014_02. Annotation GO 8340 (determination of adult lifespan) was Inferred from Mutant Phenotype

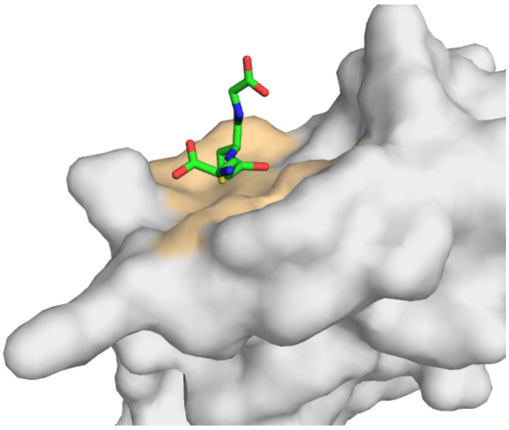

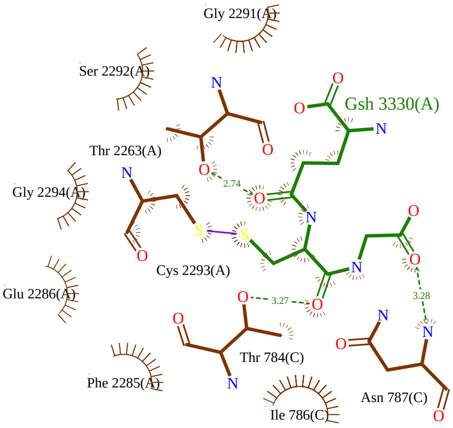

protein	amino acids contacts (binding site)						
PDB:2brq:chainA:P21333	T	F	E	G	S	C	G
tr:H0Y5C6:H0Y5C6_HUMAN	T	F	E	G	S	C	G
tr:E9PHF0:E9PHF0_HUMAN	T	F	E	G	S	C	G
tr:Q96C61:Q96C61_HUMAN	T	F	E	G	S	C	G
tr:Q6NXF2:Q6NXF2_HUMAN	T	F	E	G	S	C	G
tr:Q59H94:Q59H94_HUMAN	T	F	E	G	S	C	G
tr:Q8TES4:Q8TES4_HUMAN	T	F	E	G	S	C	G
tr:Q5HY54:Q5HY54_HUMAN	T	F	E	G	S	C	G
tr:Q60FE6:Q60FE6_HUMAN	T	F	E	G	S	C	G
tr:Q60FE5:Q60FE5_HUMAN	T	F	E	G	S	C	G
sp:P21333:FLNA_HUMAN	T	F	E	G	S	C	G
sp:Q14315:FLNC_HUMAN	T	F	E	G	S	C	G
tr:C0JPT7:C0JPT7_RAT	T	F	E	G	S	C	G
sp:D3ZHA0:FLNC_RAT	T	F	E	G	S	C	G
tr:J3JS91:J3JS91_MOUSE	T	F	E	G	S	C	G
tr:Q3UXJ8:Q3UXJ8_MOUSE	T	F	E	G	S	C	G
tr:B7FAV1:B7FAV1_MOUSE	T	F	E	G	S	C	G
tr:B7FAU9:B7FAU9_MOUSE	T	F	E	G	S	C	G
sp:Q8BTM8:FLNA_MOUSE	T	F	E	G	S	C	G
tr:D3YW87:D3YW87_MOUSE	T	F	E	G	S	C	G
sp:Q8VHX6:FLNC_MOUSE	T	F	E	G	S	C	G
tr:D3Z576:D3Z576_MOUSE	T	F	E	G	S	C	G
tr:Q6NN54:Q6NN54_DROME	T	F	K	G	S	C	D
tr:C7LAD0:C7LAD0_DROME	T	F	K	G	S	C	D
tr:A4V310:A4V310_DROME	T	F	K	G	S	C	D
tr:C7LAD1:C7LAD1_DROME	T	F	K	G	S	C	D
tr:B7Z0L2:B7Z0L2_DROME	T	F	K	G	S	C	D
tr:Q8T3K7:Q8T3K7_DROME	T	F	K	G	S	C	D
tr:Q29R34:Q29R34_DROME	T	F	K	G	S	C	D
sp:Q9VEN1:FLNA_DROME	T	F	K	G	S	C	D
tr:Q9GQV2:Q9GQV2_DROME	T	F	K	G	S	C	D
tr:Q7KSF4:Q7KSF4_DROME	T	F	K	G	S	C	D
tr:D0IMZ6:D0IMZ6_CAEEL	H	F	K	G	N	C	H
tr:Q3V5H2:Q3V5H2_CAEEL	H	F	K	G	N	C	H
tr:D0IMZ8:D0IMZ8_CAEEL	H	F	K	G	N	C	H
tr:D0IMZ7:D0IMZ7_CAEEL	H	F	K	G	N	C	H
tr:D0IMZ5:D0IMZ5_CAEEL	H	F	K	G	N	C	H

protein	whole protein ident	simil	domain-based ident	simil	contact-based ident	simil
PDB:2brq:chainA:P21333	1.0	1.0	1.0	1.0	1.0	1.0
tr:H0Y5C6:H0Y5C6_HUMAN	0.11	0.11	1.0	1.0	1.0	1.0
tr:E9PHF0:E9PHF0_HUMAN	0.29	0.29	1.0	1.0	1.0	1.0
tr:Q96C61:Q96C61_HUMAN	0.32	0.32	1.0	1.0	1.0	1.0
tr:Q6NXF2:Q6NXF2_HUMAN	0.37	0.37	1.0	1.0	1.0	1.0
tr:Q59H94:Q59H94_HUMAN	0.33	0.42	0.89	0.96	1.0	1.0
tr:Q8TES4:Q8TES4_HUMAN	0.55	0.55	1.0	1.0	1.0	1.0
tr:Q5HY54:Q5HY54_HUMAN	0.98	0.98	1.0	1.0	1.0	1.0
tr:Q60FE6:Q60FE6_HUMAN	0.99	0.99	1.0	1.0	1.0	1.0
tr:Q60FE5:Q60FE5_HUMAN	0.99	0.99	1.0	1.0	1.0	1.0
sp:P21333:FLNA_HUMAN	1.0	1.0	1.0	1.0	1.0	1.0
sp:Q14315:FLNC_HUMAN	0.7	0.88	0.89	0.96	1.0	1.0
tr:C0JPT7:C0JPT7_RAT	0.97	0.99	0.99	1.0	1.0	1.0
sp:D3ZHA0:FLNC_RAT	0.7	0.89	0.89	0.96	1.0	1.0
tr:J3JS91:J3JS91_MOUSE	0.1	0.1	0.98	0.99	1.0	1.0
tr:Q3UXJ8:Q3UXJ8_MOUSE	0.19	0.23	0.89	0.96	1.0	1.0
tr:B7FAV1:B7FAV1_MOUSE	0.95	0.97	0.98	0.99	1.0	1.0
tr:B7FAU9:B7FAU9_MOUSE	0.97	0.99	0.98	0.99	1.0	1.0
sp:Q8BTM8:FLNA_MOUSE	0.97	0.99	0.98	0.99	1.0	1.0
tr:D3YW87:D3YW87_MOUSE	0.7	0.87	0.89	0.96	1.0	1.0
sp:Q8VHX6:FLNC_MOUSE	0.7	0.89	0.89	0.96	1.0	1.0
tr:D3Z576:D3Z576_MOUSE	0.7	0.88	0.89	0.96	1.0	1.0
tr:Q6NN54:Q6NN54_DROME	0.17	0.27	0.65	0.88	0.71	0.77
tr:C7LAD0:C7LAD0_DROME	0.17	0.27	0.65	0.88	0.71	0.77
tr:A4V310:A4V310_DROME	0.17	0.27	0.65	0.88	0.71	0.77
tr:C7LAD1:C7LAD1_DROME	0.17	0.29	0.65	0.88	0.71	0.77
tr:B7Z0L2:B7Z0L2_DROME	0.17	0.29	0.65	0.88	0.71	0.77
tr:Q8T3K7:Q8T3K7_DROME	0.24	0.43	0.65	0.88	0.71	0.77
tr:Q29R34:Q29R34_DROME	0.24	0.43	0.65	0.88	0.71	0.77
sp:Q9VEN1:FLNA_DROME	0.41	0.68	0.65	0.88	0.71	0.77
tr:Q9GQV2:Q9GQV2_DROME	0.41	0.68	0.65	0.88	0.71	0.77
tr:Q7KSF4:Q7KSF4_DROME	0.43	0.74	0.65	0.88	0.71	0.77
tr:D0IMZ6:D0IMZ6_CAEEL	0.14	0.26	0.54	0.86	0.43	0.69
tr:Q3V5H2:Q3V5H2_CAEEL	0.14	0.27	0.54	0.86	0.43	0.69
tr:D0IMZ8:D0IMZ8_CAEEL	0.26	0.6	0.54	0.86	0.43	0.69
tr:D0IMZ7:D0IMZ7_CAEEL	0.26	0.6	0.54	0.86	0.43	0.69
tr:D0IMZ5:D0IMZ5_CAEEL	0.26	0.61	0.54	0.86	0.43	0.69

cher (FBgn0014141) associated phenotypes

chemical sensitive, heat sensitive, learning defective, locomotor behavior defective, long lived, male fertile, memory defective, neuroanatomy defective, short lived, uncoordinated

(Information from FlyBase)

cher (UniProt:Q9VEN1) annotation

Function: Involved in the germline ring canal formation. May tether actin microfilament within the ovarian ring canal to the cell membrane. Contributes to actin microfilaments organization. (PubMed:10477759, PubMed:10556087).
Subunit: Interacts with Ten-m. (PubMed:21857973).
Subcellular location: Cytoplasm, cytoskeleton. Cell membrane. Note=Localizes to intercellular bridges corresponding to ring canals that connect cytoplasm of nurse cells to the developing oocyte. Localizes to nurse cell plasma membranes. Detected in both the inner and outer rims of the ring canal.
Tissue specificity: Germline-specific in females (at protein level). Expressed in ovary. (PubMed:10477759, PubMed:10556087).
Developmental stage: Expressed throughout the egg-chamber development (at protein level). Expressed in the embryo. (PubMed:10477759, PubMed:10556087).
Disruption phenotype: Shows defect in ring canal assembly and female sterility. (PubMed:10477759).

(Information from UniProt)

fln-1 (WBGene00022048) associated phenotypes
reduced brood size

(Information from WormBase)

Thioredoxin-1

Best gene implication in ageing for this target family came from gene Q9V429 annotated in UniProt release 2014_02. Annotation GO 8340 (determination of adult lifespan) was Inferred from Mutant Phenotype

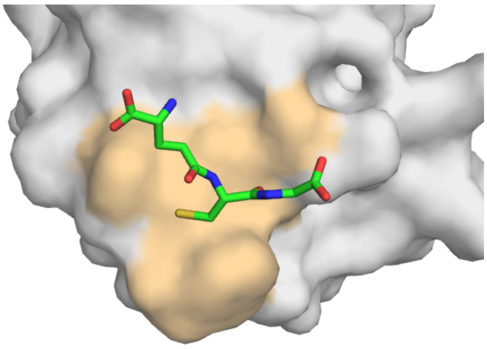

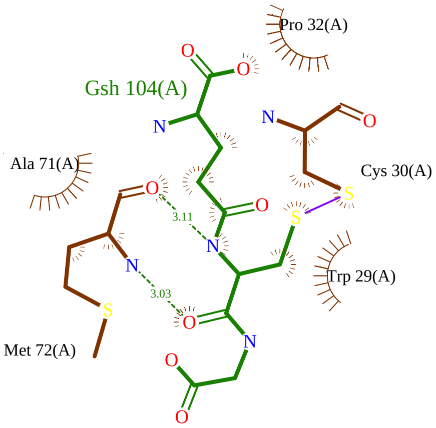

protein	amino acids contacts (binding site)				
PDB:3f3r:chainA:P22217	W	C	P	A	M
tr:B7ZME0:B7ZME0_HUMAN	R	C	P	T	I
sp:P10599:THIO_HUMAN	W	C	P	C	M
tr:H9ZYJ2:H9ZYJ2_HUMAN	W	C	P	C	M
tr:A9Z1W9:A9Z1W9_HUMAN	R	C	P	T	I
sp:Q6A555:TXND8_HUMAN	R	C	P	T	I
tr:R4GNK3:R4GNK3_RAT	W	C	P	C	M
tr:F1LV53:F1LV53_RAT	W	C	P	H	L
sp:Q5XHX6:TXND2_RAT	W	C	P	H	L
sp:P10639:THIO_MOUSE	W	C	P	C	M
sp:Q69AB2:TXND8_MOUSE	W	C	P	M	L
tr:Q14AP0:Q14AP0_MOUSE	W	C	P	M	L
tr:D3DMZ9:D3DMZ9_DROME	W	C	P	S	M
sp:P47938:THIO1_DROME	W	C	P	S	M
sp:Q9V429:THIO2_DROME	W	C	P	S	M
tr:Q9VUG9:Q9VUG9_DROME	W	C	P	S	M
sp:Q8IFW4:THIOT_DROME	W	C	P	S	M
tr:C8VV89:C8VV89_DROME	W	C	P	S	M
tr:Q9TXY8:Q9TXY8_CAEEL	W	C	P	S	M
sp:P22217:TRX1_YEAST	W	C	P	A	M
sp:P25372:TRX3_YEAST	W	C	P	A	M

protein	whole protein ident	simil	domain-based ident	simil	contact-based ident	simil
PDB:3f3r:chainA:P22217	0.96	0.97	0.99	0.99	1.0	1.0
tr:B7ZME0:B7ZME0_HUMAN	0.18	0.44	0.19	0.46	0.4	0.63
sp:P10599:THIO_HUMAN	0.45	0.79	0.47	0.8	0.8	0.55
tr:H9ZYJ2:H9ZYJ2_HUMAN	0.45	0.79	0.47	0.8	0.8	0.55
tr:A9Z1W9:A9Z1W9_HUMAN	0.18	0.48	0.2	0.5	0.4	0.63
sp:Q6A555:TXND8_HUMAN	0.23	0.62	0.24	0.62	0.4	0.63
tr:R4GNK3:R4GNK3_RAT	0.41	0.76	0.42	0.77	0.8	0.55
tr:F1LV53:F1LV53_RAT	0.06	0.15	0.32	0.76	0.6	0.77
sp:Q5XHX6:TXND2_RAT	0.06	0.14	0.32	0.76	0.6	0.77
sp:P10639:THIO_MOUSE	0.43	0.78	0.44	0.79	0.8	0.55
sp:Q69AB2:TXND8_MOUSE	0.28	0.64	0.29	0.65	0.6	0.77
tr:Q14AP0:Q14AP0_MOUSE	0.24	0.53	0.29	0.65	0.6	0.77
tr:D3DMZ9:D3DMZ9_DROME	0.42	0.81	0.42	0.81	0.8	0.77
sp:P47938:THIO1_DROME	0.29	0.75	0.29	0.77	0.8	0.77
sp:Q9V429:THIO2_DROME	0.37	0.74	0.36	0.74	0.8	0.77
tr:Q9VUG9:Q9VUG9_DROME	0.28	0.58	0.38	0.77	0.8	0.77
sp:Q8IFW4:THIOT_DROME	0.24	0.52	0.35	0.78	0.8	0.77
tr:C8VV89:C8VV89_DROME	0.22	0.48	0.36	0.78	0.8	0.77
tr:Q9TXY8:Q9TXY8_CAEEL	0.32	0.75	0.32	0.76	0.8	0.77
sp:P22217:TRX1_YEAST	1.0	1.0	1.0	1.0	1.0	1.0
sp:P25372:TRX3_YEAST	0.36	0.66	0.46	0.84	1.0	1.0

dhd (FBgn0011761) associated phenotypes
maternal effect

(Information from FlyBase)

dhd (UniProt:P47938) annotation
Function: Participates in various redox reactions through the reversible oxidation of its active center dithiol to a disulfide and catalyzes dithiol-disulfide exchange reactions. As a reducing substrate of peroxiredoxin 1, thioredoxin 2 is preferred over thioredoxin 1. Required for female meiosis and early embryonic development. (PubMed:11877442, PubMed:9106167).
Subcellular location: Nucleus (PubMed:14579129).
Tissue specificity: Ovary specific. Expressed present in the nurse cells from stage 9 of ovary development and is transported into the oocyte. Expressed throughout oogenesis. (PubMed:14579129).
Developmental stage: Expressed both maternally and zygotically. (PubMed:9106167).
Miscellaneous: The TrxT gene, which encodes an testis specific thioredoxin, is adjacent to the dhd gene and shares some regulatory region with it.

(Information from UniProt)

Trx-2 (FBgn0040070) associated phenotypes
chemical resistant, chemical sensitive, conditional, immune response defective, locomotor behavior defective, long lived, male fertile, oxidative stress response defective, short lived

(Information from FlyBase)

Trx-2 (UniProt:Q9V429) annotation
Function: Participates in various redox reactions through the reversible oxidation of its active center dithiol to a disulfide and catalyzes dithiol-disulfide exchange reactions. As a reducing substrate of peroxiredoxin 1, thioredoxin 2 is preferred over thioredoxin 1. (PubMed:11877442).
Subunit: Monomer. (PubMed:15644209).
Developmental stage: Larval stages. ECO:0000269—PubMed:11877442.

(Information from UniProt)

TrxT (FBgn0029752) associated phenotypes
chemical resistant, long lived

(Information from FlyBase)

TrxT (UniProt:Q8IFW4) annotation
Function: Probably participates in various redox reactions through the reversible oxidation of its active center dithiol to a disulfide and catalyzes dithiol-disulfide exchange reactions. Its tissue specificity suggests a regulatory role in the germline.
Subcellular location: Nucleus (PubMed:14579129). Chromosome (PubMed:14579129). Note=Specifically associated with the Y chromosome loops.
Tissue specificity: Testis specific. Not expressed in the embryo. Becomes progressively more strongly expressed during larval and pupal development. In testis, it is strongly expressed in young spermatocytes, and postmeiotic spermatid stages, then expression decreases at the nuclear elongation stage. Strongly expressed in the waste bag, in which material no longer needed for the mature sperm is eliminated. Not expressed in the stem cells and spermatogonial cells. (PubMed:14579129).
Developmental stage: Not expressed maternally. (PubMed:14579129).
Miscellaneous: The TrxT gene, which encodes an testis specific thioredoxin, is adjacent to the dhd gene and shares some regulatory region with it.

(Information from UniProt)

Integrin beta-7

Best gene implication in ageing for this target family came from gene P11584 annotated in UniProt release 2014_02. Annotation GO 8340 (determination of adult lifespan) was Inferred from Mutant Phenotype

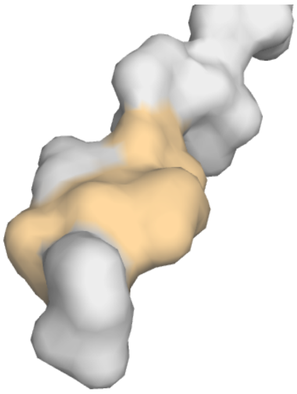

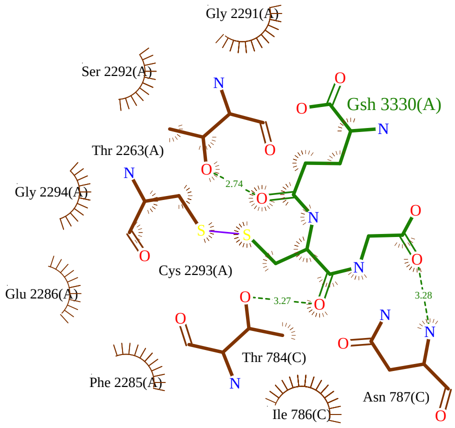

protein	amino acids contacts (binding site)		
PDB:2brq:chainC:P26010	T	I	N
tr:B7Z506:B7Z506_HUMAN	T	I	N
sp:P26010:ITB7_HUMAN	T	I	N
tr:Q4G029:Q4G029_RAT	T	V	N
tr:F7F4S8:F7F4S8_RAT	T	M	N
sp:P49134:ITB1_RAT	T	V	N
tr:G3V7M2:G3V7M2_RAT	T	V	N
tr:Q542I8:Q542I8_MOUSE	T	M	N
sp:P09055:ITB1_MOUSE	T	V	N
sp:P26011:ITB7_MOUSE	T	V	N
sp:P11584:ITBX_DROME	T	K	N
sp:Q27874:PAT3_CAEEL	T	K	N

protein	whole protein ident	simil	domain-based ident	simil	contact-based ident	simil
PDB:2brq:chainC:P26010	1.0	1.0	1.0	1.0	1.0	1.0
tr:B7Z506:B7Z506_HUMAN	0.78	0.78	1.0	1.0	1.0	1.0
sp:P26010:ITB7_HUMAN	1.0	1.0	1.0	1.0	1.0	1.0
tr:Q4G029:Q4G029_RAT	0.42	0.47	0.9	0.98	0.67	0.87
tr:F7F4S8:F7F4S8_RAT	0.41	0.74	0.51	0.82	0.67	0.95
sp:P49134:ITB1_RAT	0.42	0.76	0.49	0.86	0.67	0.87
tr:G3V7M2:G3V7M2_RAT	0.85	0.95	0.9	0.98	0.67	0.87
tr:Q542I8:Q542I8_MOUSE	0.42	0.75	0.54	0.83	0.67	0.95
sp:P09055:ITB1_MOUSE	0.42	0.76	0.49	0.86	0.67	0.87
sp:P26011:ITB7_MOUSE	0.85	0.95	0.92	0.98	0.67	0.87
sp:P11584:ITBX_DROME	0.32	0.68	0.46	0.82	0.67	0.53
sp:Q27874:PAT3_CAEEL	0.35	0.73	0.41	0.79	0.67	0.53

mys (FBgn0004657) associated phenotypes

cell shape defective, flightless, germline clone, heat sensitive, lethal - all die before end of first instar larval stage, lethal - all die before end of pupal stage, neuroanatomy defective, neurophysiology defective, partially, partially lethal - majority die, reduced, rescuable maternal effect, smell perception defective, somatic clone, some die during pupal stage

(Information from FlyBase)

mys (UniProt:P11584) annotation

Function: Integrin alpha-PS1/beta-PS is a receptor for laminin. Integrin alpha-PS2/beta-PS is a receptor for Tig, wb and Ten-m. Contributes to endodermal integrity and adhesion between the midgut epithelium and the surrounding visceral muscle. Essential for migration of the primordial midgut cells and for maintaining, but not establishing, cell polarity in the midgut epithelium. The two beta subunits mediate midgut migration by distinct mechanisms: beta-PS requires rhea/talin and Itgbetanu does not. Required for rhea/talin correct cellular localization in the midgut. Required for many embryonic (dorsal closure and somatic muscle attachments) and post-embryonic developmental processes (attachment between cell layers of imaginal disks, organization of ommatidial arrays and flight muscle development). Involved in the function and/or development of the olfactory system. Plays a role in timely border cell migration during oogenesis. (PubMed:10821184, PubMed:15469969, PubMed:19035354, PubMed:7924982, PubMed:7972082, PubMed:8119134, PubMed:9660786).
Subunit: Heterodimer of an alpha and a beta subunit. Beta-PS associates with either alpha-PS1, alpha-PS2, alpha-PS3, alpha-PS4 or alpha-PS5.
Subcellular location: Apical cell membrane (PubMed:19035354); Single-pass type I membrane protein (PubMed:19035354). Lateral cell membrane (PubMed:19035354); Single-pass type I membrane protein (PubMed:19035354). Basal cell membrane (PubMed:19035354); Single-pass type I membrane protein (PubMed:19035354). Note=In ovary, localizes to the apical, lateral and basal membranes of follicle cells through oogenesis stage 10A. Apical membrane expression peaks at oogenesis stages 9 and 10A in columnar follicle cells overlying the oocyte but decreases in the most posterior follicle cells. Thereafter, it is down-regulated. Localization to lateral and basal membranes persists during dorsal appendage morphogenesis.
Tissue specificity: In ovaries, strongly expressed in follicle cells. In oocytes, expressed in the forming dorsal appendages (at protein level). Expressed in the embryonic dorsal cuticle, the larval eye and the wing imaginal disk. (PubMed:19035354, PubMed:8119134).
Disruption phenotype: In zygotic mutant embryos, midgut forms primary constrictions but fails to elongate and the visceral muscle does not flatten but remains attached to the midgut epithelium. Embryos lacking maternal and zygotic mys show a delay in midgut migration. Mutant larvae present an olfatory phenotype, showing reduced response to isoamyl acetate but normal response to ethyl acetate. (PubMed:10821184, PubMed:15469969).
Miscellaneous: The absence of the beta-PS subunit results in detachment and rounding up of the muscles, thus the gene encoding beta-PS is called myospheroid.

(Information from UniProt)

pat-3 (WBGene00003930) associated phenotypes

body wall muscle actin organization defective, body wall muscle cell polarization variant, body wall muscle myosin organization defective, cell migration variant, embryonic lethal, excretory canal short, paralyzed arrested elongation two fold, protein expression absent, sterile

(Information from WormBase)

pat-3 (UniProt:Q27874) annotation

Function: Possible role in cell-cell interactions. Integrin alpha pat-1/beta pat-3 is a receptor for laminin. Integrin alpha pat- 2/beta pat-3 recognizes the sequence R-G-D in its ligands (By similarity). During gonad morphogenesis, involved in distal tip cell (DTC)-mediated guidance of gonad elongation, in maintaining their sharp tapering morphology and in their migration (PubMed:19023419). (, PubMed:19023419).

Subunit: Heterodimer of an alpha and a beta subunit. Beta pat-3 associates with either alpha pat-1 or alpha pat-2.

Subcellular location: Membrane ; Single-pass type I membrane protein

Disruption phenotype: RNAi-mediated knockdown in distal tip cell (DTC) causes DTC migration and guidance defects during the second phase of gonad elongation resulting in a triangular shaped gonad. (PubMed:19023419).

(Information from UniProt)

Glutamate–cysteine ligase

Best gene implication in ageing for this target family came from gene A4V448 via mapping the annotation from Ensembl FBgn0040319 via mapping the annotation from EntrezGene 53581 via mapping the annotation from GenAgeModels 0293 annotated in GenAge release 17.

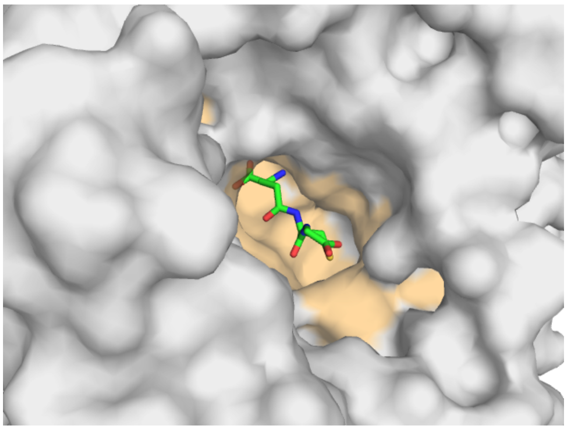

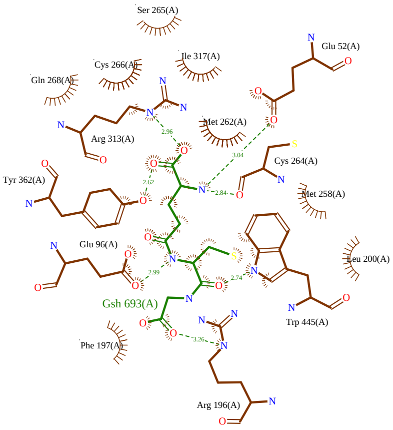

protein	amino acids contacts (binding site)
PDB:3lvw:chainA:P32477	E E R F L M M C S C Q R I Y W R
sp:P48506:GSH1_HUMAN	E E R F L M M N C C Q R I Y W R
tr:Q14TF0:Q14TF0_HUMAN	E E R F L M M N C C Q R I Y W R
sp:P19468:GSH1_RAT	E E R F L M M N C C Q R I Y W R
tr:Q3TEF1:Q3TEF1_MOUSE	E E R F L M M N C C Q R I Y W R
sp:P97494:GSH1_MOUSE	E E R F L M M N C C Q R I Y W R
tr:A4V448:A4V448_DROME	E E R F L M M C C C Q R I Y W R
sp:Q9W3K5:GSH1_DROME	E E R F L M M C C C Q R I Y W R
sp:Q20117:GSH1_CAEEL	E E R F L M M C C C Q R I Y W R
tr:E9P9G0:E9P9G0_YEASX	E E R F L M M C S C Q R I Y W R
tr:E9P9M0:E9P9M0_YEASX	E E R F L M M C S C Q R I Y W R
tr:F5CIK1:F5CIK1_YEASX	E E R F L M M C S C Q R I Y W R
sp:P32477:GSH1_YEAST	E E R F L M M C S C Q R I Y W R

	whole protein		domain-based		contact-based	
protein	ident	simil	ident	simil	ident	simil
PDB:3lvw:chainA:P32477	1.0	1.0	1.0	1.0	1.0	1.0
sp:P48506:GSH1_HUMAN	0.4	0.72	0.41	0.72	0.88	0.85
tr:Q14TF0:Q14TF0_HUMAN	0.4	0.72	0.41	0.72	0.88	0.85
sp:P19468:GSH1_RAT	0.41	0.72	0.41	0.72	0.88	0.85
tr:Q3TEF1:Q3TEF1_MOUSE	0.41	0.72	0.41	0.72	0.88	0.85
sp:P97494:GSH1_MOUSE	0.41	0.72	0.41	0.72	0.88	0.85
tr:A4V448:A4V448_DROME	0.33	0.62	0.36	0.63	0.94	0.93
sp:Q9W3K5:GSH1_DROME	0.33	0.62	0.36	0.63	0.94	0.93
sp:Q20117:GSH1_CAEEL	0.34	0.68	0.37	0.7	0.94	0.93
tr:E9P9G0:E9P9G0_YEASX	1.0	1.0	1.0	1.0	1.0	1.0
tr:E9P9M0:E9P9M0_YEASX	1.0	1.0	1.0	1.0	1.0	1.0
tr:F5CIK1:F5CIK1_YEASX	1.0	1.0	0.99	1.0	1.0	1.0
sp:P32477:GSH1_YEAST	1.0	1.0	1.0	1.0	1.0	1.0

Gclc (FBgn0040319) associated phenotypes

chemical resistant, long lived

(Information from FlyBase)

Gclc (UniProt:Q9W3K5) annotation

Pathway: Sulfur metabolism; glutathione biosynthesis; glutathione from L-cysteine and L-glutamate: step 1/2.

(Information from UniProt)

gcs-1 (UniProt:Q20117) annotation

Pathway: Sulfur metabolism; glutathione biosynthesis; glutathione from L-cysteine and L-glutamate: step 1/2.

(Information from UniProt)

Glutathione S-transferase S1

Best gene implication in ageing for this target family came from gene P41043 via mapping the annotation from Ensembl FBgn0010226 via mapping the annotation from EntrezGene 36927 via mapping the annotation from GenAgeModels 0851 annotated in GenAge release 17.

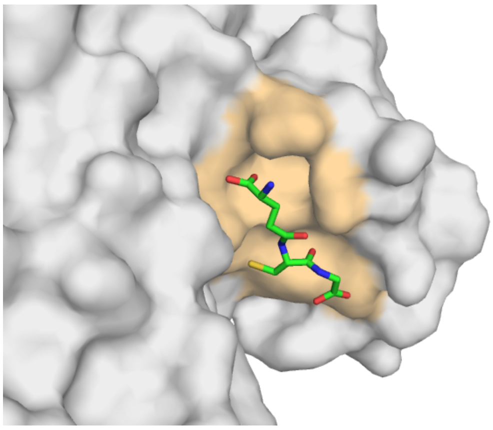

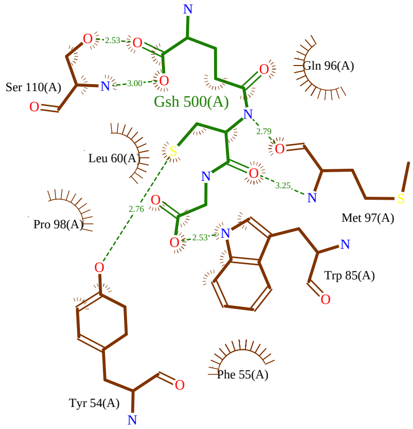

protein	amino acids contacts (binding site)
PDB:1m0u:chainA:P41043	Y F L W Q M P Q S
sp:O60760:HPGDS_HUMAN	Y F R W K I P Q S
sp:O35543:HPGDS_RAT	Y F R W K I P Q S
sp:Q9JHF7:HPGDS_MOUSE	Y F R W K I P Q S
tr:A4UZL5:A4UZL5_DROME	Y F L W Q M P Q S
sp:P41043:GST1_DROME	Y F L W Q M P Q S
tr:Q21945:Q21945_CAEEL	Y F R W K L P Q S

	whole protein		domain-based		contact-based	
protein	ident	simil	ident	simil	ident	simil
PDB:1m0u:chainA:P41043	1.0	1.0	1.0	1.0	1.0	1.0
sp:O60760:HPGDS_HUMAN	0.29	0.63	0.44	0.83	0.67	0.81
sp:O35543:HPGDS_RAT	0.31	0.64	0.47	0.84	0.67	0.81
sp:Q9JHF7:HPGDS_MOUSE	0.31	0.64	0.47	0.84	0.67	0.81
tr:A4UZL5:A4UZL5_DROME	1.0	1.0	1.0	1.0	1.0	1.0
sp:P41043:GST1_DROME	1.0	1.0	1.0	1.0	1.0	1.0
tr:Q21945:Q21945_CAEEL	0.27	0.62	0.44	0.81	0.67	0.8

GstS1 (FBgn0010226) associated phenotypes

partially lethal - majority live

(Information from FlyBase)

GstS1 (UniProt:P41043) annotation

Function: Conjugation of reduced glutathione to a wide number of exogenous and endogenous hydrophobic electrophiles. May be involved in the detoxification of metabolites produced during cellular division and morphogenesis.
Subunit: Homodimer. ECO:0000269—PubMed:12547198.
Developmental stage: Expressed throughout development with highest levels being observed in nonfeeding stages, i.e. During embryonic and pupal development.

(Information from UniProt)

Glutathione synthetase

Best gene implication in ageing for this target family came from gene P46413 annotated in UniProt release 2014_02. Annotation GO 7568 (aging) was Inferred from Expression Pattern

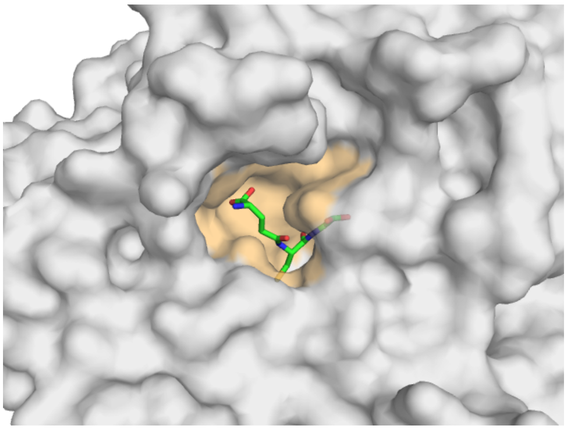

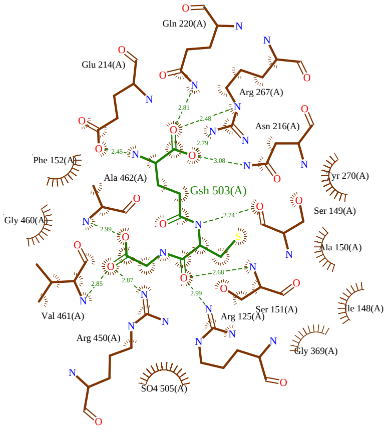

protein	amino acids contacts (binding site)
PDB:2hgs:chainA:P48637	R I S A S F E N Q R Y G R G V A
tr:B7Z1C5:B7Z1C5_HUMAN	R I S A S F E N Q R Y G R G V A
sp:P48637:GSHB_HUMAN	R I S A S F E N Q R Y G R G V A
sp:P46413:GSHB_RAT	R I S A S F E N Q R Y G R G V A
tr:Q3UEE2:Q3UEE2_MOUSE	R I S A S F E N Q R Y G R G V A
sp:P51855:GSHB_MOUSE	R I S A S F E N Q R Y G R G V A
tr:Q541E2:Q541E2_MOUSE	R I S A S F E N Q R Y G R G V A
tr:Q8IQZ1:Q8IQZ1_DROME	R V A S S F S N Q R Y G R G V A
tr:A3FP35:A3FP35_DROME	R V A S S F S N Q R Y G R G V A
tr:Q86B44:Q86B44_DROME	R I A S G F S N Q R Y G R G V A
tr:Q21549:Q21549_CAEEL	R I A S S M N N Q R Y G R G V A
tr:E9P9G1:E9P9G1_YEASX	R V S V S F E N Q R Y G R G V A
tr:E9P9L9:E9P9L9_YEASX	R V S V S F E N Q R Y G R G V A
sp:Q08220:GSHB_YEAST	R V S V S F E N Q R Y G R G V A

protein	whole protein ident	whole protein simil	domain-based ident	domain-based simil	contact-based ident	contact-based simil
PDB:2hgs:chainA:P48637	1.0	1.0	1.0	1.0	1.0	1.0
tr:B7Z1C5:B7Z1C5_HUMAN	0.85	0.85	1.0	1.0	1.0	1.0
sp:P48637:GSHB_HUMAN	1.0	1.0	1.0	1.0	1.0	1.0
sp:P46413:GSHB_RAT	0.88	0.97	0.9	0.97	1.0	1.0
tr:Q3UEE2:Q3UEE2_MOUSE	0.77	0.83	0.9	0.97	1.0	1.0
sp:P51855:GSHB_MOUSE	0.89	0.97	0.9	0.97	1.0	1.0
tr:Q541E2:Q541E2_MOUSE	0.89	0.97	0.9	0.97	1.0	1.0
tr:Q8IQZ1:Q8IQZ1_DROME	0.4	0.76	0.43	0.79	0.75	0.82
tr:A3FP35:A3FP35_DROME	0.4	0.77	0.43	0.79	0.75	0.82
tr:Q86B44:Q86B44_DROME	0.35	0.67	0.35	0.64	0.75	0.81
tr:Q21549:Q21549_CAEEL	0.38	0.75	0.4	0.76	0.75	0.84
tr:E9P9G1:E9P9G1_YEASX	0.34	0.72	0.38	0.74	0.88	0.95
tr:E9P9L9:E9P9L9_YEASX	0.34	0.72	0.38	0.74	0.88	0.95
sp:Q08220:GSHB_YEAST	0.34	0.72	0.38	0.74	0.88	0.95

GS (UniProt:Q8IQZ1) annotation

Cofactor: Mg(2+) Evidence=(PIRNR:PIRNR001558); Note=Binds 1 Mg(2+) ion per subunit. (PIRNR:PIRNR001558);
Pathway: Sulfur metabolism; glutathione biosynthesis; glutathione from L-cysteine and L-glutamate: step 2/2. (PIRNR:PIRNR001558).

(Information from UniProt)

UniProt:A3FP35 annotation

Cofactor: Mg(2+) Evidence=(PIRNR:PIRNR001558, PIRSR:PIRSR001558-2); Note=Binds 1 Mg(2+) ion per subunit. (PIRNR:PIRNR001558, PIRSR:PIRSR001558-2);
Pathway: Sulfur metabolism; glutathione biosynthesis; glutathione from L-cysteine and L-glutamate:

step 2/2. (PIRNR:PIRNR001558).

(Information from UniProt)

gss-1 (WBGene00010941) associated phenotypes

lethal, sterile

(Information from WormBase)

gss-1 (UniProt:Q21549) annotation

Cofactor: Mg(2+) Evidence=(PIRNR:PIRNR001558, PIRSR:PIRSR001558-2); Note=Binds 1 Mg(2+) ion per subunit. (PIRNR:PIRNR001558, PIRSR:PIRSR001558-2);
Pathway: Sulfur metabolism; glutathione biosynthesis; glutathione from L-cysteine and L-glutamate: step 2/2. (PIRNR:PIRNR001558).

(Information from UniProt)

Probable glutathione S-transferase 5

Best gene implication in ageing for this target family came from gene Q09596 via mapping the annotation from WormBase WBGene00001753 annotated in WormBase 2014-03-11. Annotation GO 8340 (determination of adult lifespan) was Inferred from Mutant Phenotype

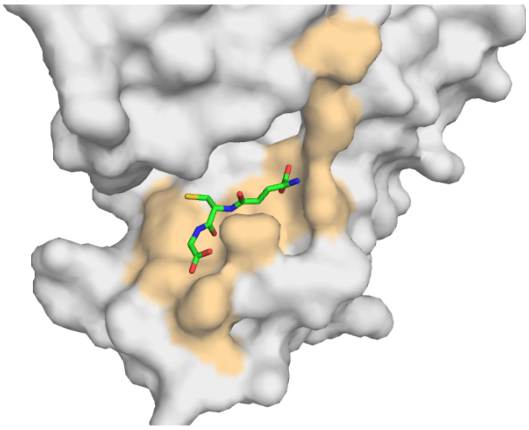

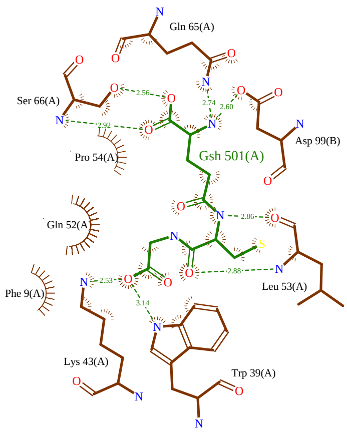

protein	amino acids contacts (binding site)
PDB:1zl9:chainA:Q09596	F W K Q L P Q S K D
sp:Q09596:GST5_CAEEL	F W K Q L P Q S K D

	whole protein		domain-based		contact-based	
protein	ident	simil	ident	simil	ident	simil
PDB:1zl9:chainA:Q09596	1.0	1.0	1.0	1.0	1.0	1.0
sp:Q09596:GST5_CAEEL	1.0	1.0	1.0	1.0	1.0	1.0

gst-5 (UniProt:Q09596) annotation

Function: Conjugation of reduced glutathione to a wide number of exogenous and endogenous hydrophobic electrophiles.

(Information from UniProt)
